# Can Voice Parameters Provide Cutoff Values to Predict Dysphagia in Individuals with Multiple Sclerosis?

**DOI:** 10.1007/s00455-024-10720-3

**Published:** 2024-06-13

**Authors:** Merve Sapmaz Atalar, Gençer Genç, Elif Ezgi Işık, Mehmet Emrah Cangi, Beyza Pehlivan, Serpil Bulut

**Affiliations:** 1 Department of Speech and Language Therapy, Hamidiye Faculty of Health Science, University of Health Sciences, İstanbul, Türkiye; 2 Department of Neurology, Şişli Hamidiye Etfal Training and Research Hospital, University of Health Sciences, İstanbul, Türkiye

**Keywords:** Multiple sclerosis, Dysphagia, Swallowing, Voice parameters, Diagnostic accuracy

## Abstract

In dysphagia assessment, along with well-defined measurements and signs, voice parameters can potentially support clinical decision as a marker, but more evidence is needed. This study aims to determine the voice parameters that can predict the risk of dysphagia and to determine optimal cutoff values in individuals with multiple sclerosis (IwMS). Seventy-six adults participated in the study, including 39 IwMS and 37 healthy individuals (HI). The study used the Dysphagia in Multiple Sclerosis Questionnaire (DYMUS), Gugging Swallowing Screen (GUSS), and Voice Handicap Index (VHI-10) and recorded voice samples using Praat programme. Voice recordings were taken pre- and post-swallowing. The voice parameters analysed are fundamental frequency (F0), standard deviation F0 (SD F0), jitter (local), shimmer (local), and harmonic-to-noise ratio (HNR). Roc analysis was performed to examine the diagnostic accuracy performance of the risk for dysphagia/penetration. The parameters of IwMS pre-swallowing differed significantly from those of HI on the VHI-10, DYMUS, GUSS scores, and jitter (local), shimmer (local), and HNR. IwMS but not HI exhibited significant differences in shimmer (local) and HNR between the pre- and post-swallowing measurements. In IwMS, GUSS revealed significant differences in shimmer (local) pre- and post-swallowing between the groups with and without dysphagia/penetration. In the ROC analysis results, the area under the curve (AUC) for shimmer (local) pre-swallowing was 73.1% (cutoff = 1.69); post-swallowing, it was 78.6% (cutoff = 1.57). In conclusion, IwMS can be associated with differences in shimmer (local) and HNR parameters, low quality of life-related to voice, and dysphagia/penetration risk. The AUC values for shimmer (local) in IwMS pre- and post-swallowing may help to strengthen diagnostic decisions of dysphagia risk.

## Introduction

Multiple sclerosis is a disease that affects the central nervous system due to damage to myelin sheaths [1]. Common symptoms of the disease include spasticity, balance and mobility problems, depression, fatigue, and voice and swallowing disorders [2–4]. In IwMS, voice impairment is characterized by a harsh, breathy voice quality and by variability in the fundamental frequency (F0) and sound pressure level [5, 6]. Impaired respiratory support can decrease maximum phonation time and the length of phrases produced with one breath. Vocal fatigue, higher jitter and shimmer values, phonation instability, and failure to maintain the adductor power required for phonation have also been reported [6–9].

IwMS also exhibit different characteristics in relation to the swallowing function and dysphagia symptoms. IwMS display have various problems with swallowing functions, including weak tongue base retraction, delayed and prolonged pharyngeal phase, pharyngeal constrictor dysmotility, a lack of laryngeal closure and impaired upper esophageal sphincter opening [10–13]. The most common symptoms of dysphagia reported by IwMS include the feeling of getting stuck in the throat, coughing and throat clearing. Another typical symptom is choking during or after and eating/drinking [14]. Dysphagia can lead to malnutrition, dehydration, aspiration pneumonia, and death, thereby reducing quality of life not only physically but also psychologically and socially [15–17]. The early diagnosis of dysphagia is crucial to reduce and prevent its complications [18].

There is a considerable overlap in the anatomical structures and functions related to the voice and swallowing. Both functions involve voluntary control of cortical and subcortical structures as well as brainstem and midbrain control systems. These functions involve complex systems that require the integration of oral, pharyngeal, laryngeal, and respiratory muscles in the upper airway. Therefore, impairments in these functions also have significant overlaps [19].

Individuals with dysphagia may have difficulty with any one or more of the anatomical and physiological components of the swallowing stages. In functional oropharyngeal disorders, reduced pharyngeal peristalsis and pressure and coordination problems due to delays in the swallowing reflex may cause residue to form in and near the vocal folds [20]. Residual material can affect the vibration of the folds and voice quality [21]. It can also affect the movement of the vocal folds and alter the period and amplitude pattern [21].

In clinical evaluations, acoustic analyses that include voice parameters can be conducted to objectively assess dysphagic patients. It has been reported that voice parameters can detect possible penetration or aspiration in acoustic analyses performed pre- and post-swallowing [21, 22]. Previous researchers have examined specific groups such as individuals with stroke, Amyotrophic lateral sclerosis, Parkinson’s disease, and head and neck cancers, and the results were varied. One study examining the relationship between voice quality and swallowing function found that a higher F0 was significantly associated with decreased oropharyngeal swallowing efficiency [23]. Conversely, in another study, it was reported that there was no significant difference in the F0 parameter after swallowing in the group with high aspiration risk compared to the group with low aspiration risk, while the shimmer percentage, voice turbulence index (VTI), noise-harmonic ratio (NHR), and relative average perturbation (RAP) parameters increased significantly [22]. In a study examining groups with and without aspiration risk factors, a significant difference was found solely for the RAP parameter [21]. In contrast to other studies, one study found that the acoustic measurements used to detect aspiration in individuals with dysphagia were not sufficiently sensitive or specific enough [24]. The inconsistency between the results of the studies and the lack of adequate studies to examine the relationship between acoustic voice parameters and dysphagia risks in IwMS prompted the execution of this study.

### Purpose

As mentioned above, dysphagia is a potential issue for IwMS and requires sensitive and specific measurements to be identified. As in the general population, clinical swallowing assessments and instrumental measurements can be employed for IwMS [3]. Clinical assessments, however, are insufficient in terms of sensitivity and accuracy of diagnosis when employed alone. Penetration, aspiration, or silent aspiration may be overlooked in clinical evaluations when auditory and visual perceptual methods are applied [21]. In light of this, it may be wise for this group to employ instrumental assessments. However, access to instrumental assessments may be limited due to the high cost of the devices. For this reason, it is believed that determining voice parameters that can predict the risk of dysphagia through relatively more accessible, simple, and non-invasive voice analysis measurements in clinics may contribute to clinical swallowing evaluations in IwMS. However, this does not change the indispensability of instrumental assessments for valid and reliable measurements.

The aim of this study was to examine the relationship between voice and swallowing and to identify voice parameters that may predict the risk of dysphagia by using ROC analysis in IwMS.

1. Is there a significant difference between IwMS and HI groups in terms of voice handicap, dysphagia risk, and pre- and post-swallowing voice parameters?
2. Is there a significant relationship between voice handicap, dysphagia risk, and pre- and post-swallowing voice parameters in IwMS and HI groups?
3. Is there a significant difference between pre- and post-swallowing voice parameters in IwMS and HI groups?
4. Is there a significant difference between acoustic voice parameters pre- and post-swallowing according to dysphagia risk score in IwMS and HI?
5. Can the risk of dysphagia be distinguished in ROC analysis with voice parameters?
6. In ROC analysis, if significance is found for any parameter, what are the specificity, sensitivity ratings and cutoff values for the parameter(s) found significant?

## Method

### Participants

The study was conducted in the Neurology Outpatient Clinic of Istanbul Sisli Hamidiye Etfal Training and Research Hospital with a total of 76 adult participants, including 39 individuals diagnosed with MS according to McDonald criteria and 37 HI [25]. The diagnosis of this disease was made by neurologists. Healthy participants were included according to their answers to the questions in the demographic information form. The inclusion criteria for IwMS were to be between the ages of 18–65, to be diagnosed with MS, and to score 24 or higher on the Mini Mental State Examination (MMSE). The inclusion criteria for IwMS and HI were to be between the ages of 18–65, and to score 24 or higher on the MMSE. Exclusion criteria for IwMS are having an additional neurological or psychiatric disease other than MS diagnosis, having any voice-related complaints, having received voice, swallowing or speech therapy in the last 6 months, and having undergone vocal fold surgery. Exclusion criteria for HI are having a neurological or psychiatric disease, having any voice-related complaints, having received voice, swallowing or speech therapy in the last 6 months, and undergoing vocal fold surgery. For the study, 50 IwMS and 46 HI completed a voluntary consent form. However, considering the inclusion criteria, 20 individuals who scored less than 24 points on the MMSE, who were diagnosed with additional neurologic diseases and who did not volunteer to participate in the study were excluded from the study.

Within the scope of the study, a total of 76 participants were included in the evaluation, of which 67.1% (51 participants) were female and 32.9% (25 participants) were male. Of the participants, 51.3% (39) were IwMS and 48.7% (37) were HI. The ages of the participants ranged from 20 to 65 years (Table 1).

**Table 1:** Distribution of Demographic Information and Clinical Results of the Groups

Characteristics	IwMS (*n* = 39)	HI (*n* = 37)
	Mean (Min-Max)	Mean (Min-Max)
Age (year)	39.6 (20–65)	38.3 (22–57)
**Gender**		
Female	28 (71.8%)	23 (62.2%)
Male	11 (28.2%)	14 (37.8%)
Disease Duration (year)	6.1 (1–17)	-
EDSS	1.6 (0–6)	-
MMSE	28.8 (25–30)	28.8 (24–30)

*Note*. IwMS = individual with Multiple sclerosis; HI = healthy individuals; Min = minimum; Max = maximum; EDSS = Kurtzke Expanded Disability Status Scale; MMSE = Mini Mental State Examination

All participant approved a voluntary consent form, and the study was carried out in compliance with the Declaration of Helsinki Principles. The University of Health Sciences-Türkiye, Hamidiye Scientific Research Ethics Committee also gave the study their approval (22/438).

### Data Collection Tools

#### Demographic Information Form

This form, which was prepared for inclusion criteria, includes demographic information including age, gender, education level and occupation. In the medical history section, the type of MS, duration of the disease, Kurtzke Expanded Disability Status Scale (EDSS) score, level of independence, medication use, additional health problems and previous surgery, if any, were all documented [26]. The MMSE was used to determine the inclusion criteria. Some data recorded in the demographic information form are presented in Table 1.

#### Mini Mental State Examination (MMSE)

The total score of the MMSE, which is used as a cognitive screening test, is 30. A score of 24 and above is defined as normal cognitive status. The Turkish version of the MMSE, for which validity and reliability study was conducted, was used in this study [27, 28].

#### Voice Handicap Index (VHI-10)

It is a short-form 10-item patient-response scale designed to assess all types of voice disorders and can be scored quickly during the assessment. The scale ranges from 0 (best) to 40 (worst). The Turkish version of the VHI-10, for which validity and reliability study was conducted, was used in this study [29, 30].

#### Dysphagia in Multiple Sclerosis Questionnaire (DYMUS)

DYMUS is a reliable and consistent scale that can be easily applied to assess oropharyngeal dysphagia in IwMS [31]. The ten-item scale, on which yes or no responses are given, yields a score between 0 and 10, and a rise in the score denotes a dysphagia-related problem. The severity of dysphagia is determined by a score of three or higher [32].

#### Gugging Swallow Screening Test (GUSS)

It is one of the bedside swallowing screening tests and is recommended for use in clinical practice to determine the risk of dysphagia [33]. GUSS has also been recommended for dysphagia assessment of IwMS [34]. The GUSS consists of two stages. The first part consists of an indirect swallowing test that evaluates the ability to maintain wakefulness, cough and/or clear the throat voluntarily at least twice, and swallow saliva. The second part consists of a direct swallowing test evaluated with semi-solid, liquid and solid foods [33, 35, 36]. On the scale, higher scores indicate a lower risk of dysphagia, while lower values indicate a higher risk. The GUSS cutoff score for “Dysphagia/penetration” is ≤ 19 and the cutoff score for “Aspiration” is ≤ 14 [37]. The Turkish version of the GUSS, for which validity and reliability study was conducted, was used in this study [36].

#### Acoustic Voice Analysis

The acoustic analysis used in this study includes voice parameters related to frequency and intensity, voice spectrography, vocal perturbation and noise. The voice parameters analysed are F0, SD F0, jitter (local), shimmer (local), and HNR. The vocal folds’ rate of vibration is known as the F0. SD F0 reflects frequency variability. Abnormal frequency variability may be caused by an organic, functional or neurological problem [38]. Period perturbations are measured based on jitter, while amplitude perturbations are measured based on shimmer [39]. HNR is the ratio of the energy in the F0 and its harmonics to the energy in the aperiodic components of the voice signal [40]. One or more of these voice parameters may be impacted by material present in the vocal folds.

### Procedure

In the first step of the study, an identical procedure was applied to all the participants after they provided written informed consent. The participants completed a demographic information form, after which the authors administered the MMSE. The participants then completed the DYMUS and VHI-10. In the second step, a baseline pre-swallowing voice recording was obtained from the participants for acoustic analysis. For acoustic voice analysis, the signal-to-noise ratio (SNR) was measured in a quiet room to measure whether environmental noise affects acoustic voice quality. All signals were acquired according to the standards recommended in the literature [41]. In line with the literature, the microphone was placed at an angle of approximately 45°-90° and a microphone-to-mouth distance of 4–10 cm, with the person in a sitting position [42]. Each participant was first asked to produce a prolonged /a/ vowel for more than 3 s with a comfortable loudness and pitch pre- and post-swallowing. Three seconds of the phonation duration were analyzed after the first and last portions of the recordings were clipped [43]. All audio recordings were recorded with a sample rate of 44.1 kHz and 16-bit resolution. Audio-Technica ATR2100 USB Cardioid Dynamic USB/XLR Microphone (Audio-Technica U.S., Inc.) was used for voice recordings [44]. The audio recordings were saved in “wav” format and transferred to the Praat program [45]. Praat (version 6.2.01) and Audacity (3.1.2 software package) were used [45, 46]. After the recording, the GUSS was administered to the participants. A second voice recording was taken after swallowing. The GUSS was scored by two speech-language therapists.

### Statistical Analysis

Data were analyzed with the IBM Statistical Package for the Social Sciences (SPSS) for Windows 23.0 (IBM Corp., Armonk, NY). The descriptive values for both continuous and categorical data were presented. The Kolmogorov-Smirnov Test was used to check whether the data are normally distributed. In intergroup comparisons, Independent Sample T-Test was used for the two groups with normal distribution and Mann Whitney-U Test was used for the ones without normal distribution. Chi-Square Test or Fisher’s Exact Test was used for comparisons of categorical variables between groups. Spearman’s Correlation Test was utilized to determine the relationship between the scale scores. In the evaluation of acoustic voice parameters pre- and post-swallowing, “Paired Sample T-Test” was used for those with normal distribution and “Wilcoxon Test” was used for those without normal distribution. ROC analysis was performed for the parameters that were thought to have a discriminative effect for the development of dysphagia/penetration and the ROC curve was drawn. Results were considered statistically significant when the p value was less than 0.05. The correlation coefficient is very low between 0.00 and 0.19, low between 0.20 and 0.49, moderate between 0.50 and 0.69, high between 0.70 and 0.89 and between 0.90 and 1.00, indicating that the relationship between the two variables is very high [47].

## Results

In accordance with the study’s purposes, a comparison was made between the swallowing assessment scores and acoustic voice parameters from IwMS and HI. There was a significant difference between the groups in DYMUS, VHI-10, GUSS, jitter (local), shimmer (local), HNR pre-swallowing and shimmer (local), and HNR post-swallowing (*p* < 0.05). It was determined that the values of the IwMS were significant higher than the HI in all parameters except HNR measurement pre- and post-swallowing (Table 2).

**Table 2:** Comparing of Groups Based on Their Voice and Swallowing Measurements

Measurements	IwMS (*n* = 39)	HI (*n* = 37)	*p*-value
	*n* (%) or (Median (Min-Max)	*n* (%) or (Median (Min-Max)	
DYMUS	0 (0–29)	0 (0–5)	**0.003**
VHI-10	2 (0–29)	0 (0–10)	**0.008**
GUSS	18 (13–20)	20 (18–20)	**< 0.001**
Pre-swallowing F0	207.69 (114.605-274.062)	224.242 (85.47-277.47)	0.571
Pre-swallowing SD F0	1.481 (0.734–4.282)	1.378 (0.485–3.165)	0.296
Pre-swallowing jitter (local)	0.287 (0.102–1.001)	0.228 (0.124–0.626)	**0.019**
Pre-swallowing shimmer (local)	1.877 (0.547–4.93)	1.185 (0.742–2.935)	**< 0.001**
Pre-swallowing HNR	24.321 (15.886–29.745)	26.081 (19.065–32.218)	**< 0.001**
Post-swallowing F0	214.121 (95.266-273.092)	217.637 (75.829-270.053)	0.651
Post-swallowing SD F0	1.639 (0.491–4.792)	1.515 (0.563–3.533)	0.277
Post-swallowing jitter (local)	0.266 (0.116–0.922)	0.222 (0.102–0.692)	0.113
Post-swallowing shimmer (local)	2.352 (0.93–8.65)	1.226 (0.626–2.506)	**< 0.001**
Post-swallowing HNR	21.263 (14.23–28.39)	25.003 (18.45-34.428)	**< 0.001**

*Note*. IwMS = individual with Multiple sclerosis; HI = healthy individuals; Min = minimum; Max = maximum; DYMUS = Dysphagia Assessment Scale in Multiple Sclerosis; VHI-10 = Voice Handicap Index; GUSS = Gugging Swallow Screening Test; F0 = fundamental frequency; SD F0 = standard deviation F0; HNR = harmonic-to-noise ratio

Table 3 shows the results of Spearman’s correlation study, which examined the correlation between the scale scores of the groups. The relationship between DYMUS scores and VHI-10 scores of IwMS was found to be significant, positive and at low level (*r* = 0.443, *p* = 0.005); the relationship between DYMUS scores and GUSS scores was found to be significant, negative and at low level (*r* = − 0.338, *p* = 0.035). The relationship between DYMUS scores and VHI-10 scores of HI was found to be significant, positive and moderate (*r* = 0.540, *p* = 0.001). The relationship between DYMUS scores and GUSS scores of HI was not found to be significant (*r*=-0.010, *p* = 0.951). The relationship between GUSS scores and VHI-10 scores of IwMS and HI was not found to be significant (*p* = 0.153; *p* = 0.625, respectively). The relationship between GUSS scores and pre-swallowing jitter (local), and post-swallowing shimmer (local) scores of IwMS was found to be significant, negative and low (*r*=-0.344, *p* = 0.032; *r*=-0.398, *p* = 0.012, respectively). The relationship between GUSS scores and pre-swallowing HNR scores of IwMS was found to be significant, positive and low (*r* = 0.379, *p* = 0.017). The relationship between DYMUS scores and pre-swallowing HNR scores of IwMS was found to be significant, negative and low (*r*=-0.321, *p* = 0.046). The relationship between GUSS scores and post-swallowing HNR scores of HI was found to be significant, positive and low (*r* = 0.389, *p* = 0.017).

**Table 3:** Correlations and Comparisons Between Voice and Swallowing Measurements in Groups

Spearman’s Correlation			GUSS	DYMUS
**IwMS**	VHI-10	*r*	-0.233	0.443^**^
		*p*	0.153	**0.005**
	GUSS	*r*	1.000	− 0.338*
		*p*	-	**0.035**
	Pre-swallowing F0	*r*	-0.032	0.075
		*p*	0.848	0.651
	Pre-swallowing SD F0	*r*	-0.052	0.198
		*p*	0.755	0.226
	Pre-swallowing jitter (local)	*r*	− 0.344^*^	0.288
		*p*	**0,032**	0.076
	Pre-swallowing shimmer (local)	*r*	-0.304	0.010
		*p*	0.060	0.950
	Pre-swallowing HNR	*r*	0.379^*^	− 0.321^*^
		*p*	**0.017**	**0.046**
	Post-swallowing F0	*r*	-0.019	0.135
		*p*	0.909	0.412
	Post-swallowing SD F0	*r*	-0.055	0.141
		*p*	0.741	0.392
	Post-swallowing jitter (local)	*r*	-0.128	0.156
		*p*	0.439	0.342
	Post-swallowing shimmer (local)	*r*	− 0.398^*^	0.247
		*p*	**0.012**	0.129
	Post-swallowing HNR	*r*	0.186	-0.241
		*p*	0.257	0.140
**HI**	VHI-10	*r*	0.083	0.540^**^
		*p*	0.625	**0.001**
	GUSS	*r*	1.000	-0.010
		*p*	-	0.951
	Pre-swallowing F0	*r*	-0.236	0.244
		*p*	0.160	0.146
	Pre-swallowing SD F0	*r*	-0.138	-0.084
		*p*	0.416	0.622
	Pre-swallowing jitter (local)	*r*	0.079	-0.292
		*p*	0.642	0.080
	Pre-swallowing shimmer (local)	*r*	-0.053	0.083
		*p*	0.754	0.625
	Pre-swallowing HNR	*r*	0.097	-0.242
		*p*	0.569	0.149
	Post-swallowing F0	*r*	-0.241	0.287
		*p*	0.151	0.085
	Post-swallowing SD F0	*r*	-0.118	0.115
		*p*	0.487	0.499
	Post-swallowing jitter (local)	*r*	0.038	-0.319
		*p*	0.824	0.055
	Post-swallowing shimmer (local)	*r*	0.070	0.162
		*p*	0.681	0.339
	Post-swallowing HNR	*r*	0.389^*^	-0.274
		*p*	**0.017**	0.101

*Note*. IwMS = individuals with multiple sclerosis; HI = healty individuals; VHI-10 = voice handicap index; GUSS = Gugging Swallow Screening Test; DYMUS = Dysphagia Assessment Scale in Multiple Sclerosis; F0 = fundamental frequency; SD F0 = standard deviation F0; HNR = harmonic-to-noise ratio

It was determined that there was a statistically significant difference in the shimmer (local) and HNR measurement values between the pre-swallowing and post-swallowing groups of IwMS (*p* < 0.05) (Table 4). However, no significant difference was detected in any measurement values pre- and post-swallowing in HI (*p* > 0.05) (Table 5).

**Table 4:** Comparison of Acoustic Voice Parameters of IwMS Pre and Post Swallowing

Acoustic Voice Parameters	Pre-swallowing	Post-swallowing	*p*-value
	Median (Min-Max)	Median (Min-Max)	
F0	207.69 (114.605-274.062)	214.121 (95.266-273.092)	0.759
SD F0	1.481 (0.734–4.282)	1.639 (0.491–4.792)	0.364
jitter (local)	0.287 (0.102–1.001)	0.266 (0.116–0.922)	0.472
shimmer (local)	**1.877 (0.547–4.93)**	**2.352 (0.93–8.65)**	**0.009**
HNR	**24.321 (15.886–29.745)**	**21.263 (14.23–28.39)**	**< 0.001**

*Note*. IwMS = individual with Multiple sclerosis; Min = minimum; Max = maximum; F0 = fundamental frequency; SD F0 = standard deviation F0; HNR = harmonic-to-noise ratio

**Table 5:** Comparison of Acoustic Voice Parameters Pre and Post Swallowing in HI

Acoustic Voice Parameters	Pre-swallowing	Post-swallowing	*p*-value
	Median (Min-Max)	Median (Min-Max)	
F0	202.00 (85.47-277.47)	198.25 (75.83-270.05)	0.054
SD F0	1.446 (0.485–3.165)	1.580 (0.563–3.533)	0.093
jitter (local)	0.245 (0.124–0.626)	0.292 (0.102–0.692)	0.091
shimmer (local)	1.301 (0.742–2.935)	1.315 (0.626–2.506)	0.592
HNR	26.296 (19.065–32.218)	25.818 (18.450-34.428)	0.455

*Note*. HI = healthy individuals; Min = minimum; Max = maximum; F0 = fundamental frequency; SD F0 = standard deviation F0; HNR = harmonic-to-noise ratio

There was no statistically significant difference in any of the pre-swallowing and post-swallowing measurements between the dysphagia risk group (DYMUS ≥ 3) and the group without dysphagia risk (DYMUS < 3) determined according to the DYMUS score in IwMS (*p* > 0.05) (Table 6). There is a statistically significant difference (*p* < 0.05) in the pre-swallowing and post-swallowing shimmer (local) measurement value between the group with (GUSS ≤ 19) and without (GUSS > 19) dysphagia/penetration risk determined according to the GUSS score in IwMS (Table 7).

**Table 6:** Comparison of Acoustic Voice Parameters Pre and Post Swallowing According to DYMUS Score in IwMS

Characteristics	No risk of dysphagia (DYMUS < 3) (*n* = 28)	Risk of dysphagia (DYMUS≥3) (*n* = 11)	*p*-value
	Median (Min-Max)	Median (Min-Max)	
Pre-swallowing F0	215.487 (115.473-274.062)	200.61 (114.605-236.914)	0.224
Pre-swallowing SD F0	1.49 (0.734–3.574)	1.481 (0.901–4.282)	0.435
Pre-swallowing jitter (local)	0.264 (0.102–1.001)	0.287 (0.169–0.456)	0.791
Pre-swallowing shimmer (local)	1.93 (0.547–3.913)	1.571 (0.896–4.93)	0.950
Pre-swallowing HNR	24.45 (15.886–29.745)	23.495 (16.071–28.723)	0.473
Post-swallowing F0	214.38 (95.266-273.092)	204.542 (105.196-241.794)	0.473
Post-swallowing SD F0	1.605 (0.491–4.792)	1.82 (0.662–4.511)	0.685
Post-swallowing jitter (local)	0.323 (0.116–0.662)	0.262 (0.213–0.922)	0.988
Post-swallowing shimmer (local)	2.247 (1.113–4.91)	2.886 (0.93–8.65)	0.275
Post-swallowing HNR	21.453 (14.23–28.39)	19.08 (14.77-24.271)	0.201

*Note*. IwMS = individual with Multiple sclerosis; Min = minimum; Max = maximum; DYMUS = Dysphagia Assessment Scale in Multiple Sclerosis; F0 = fundamental frequency; SD F0 = standard deviation F0; HNR = harmonic-to-noise ratio

**Table 7:** Comparison of Acoustic Voice Parameters Pre and Post Swallowing According to GUSS Score in IwMS

Characteristics	No dysphagia/penetration (GUSS > 19) (*n* = 10)	Dysphagia/penetration is present (GUSS ≤ 19) (*n* = 29)	*p*-value
	Median (Min-Max)	Median (Min-Max)	
Pre-swallowing F0	210.02 (115.473–261.87)	207.69 (114.605-274.062)	0.974
Pre-swallowing SD F0	1.684 (0.744–2.631)	1.461 (0.734–4.282)	0.772
Pre-swallowing jitter (local)	0.239 (0.102–0.346)	0.288 (0.141–1.001)	0.157
Pre-swallowing shimmer (local)	**1.394 (0.547–2.514)**	**2.097 (0.828–4.93)**	**0.031**
Pre-swallowing HNR	25.653 (21.714–29.745)	24.146 (15.886–28.723)	0.067
Post-swallowing F0	210.768 (117.413-266.652)	214.121 (95.266-273.092)	0.748
Post-swallowing SD F0	1.559 (1.005–4.511)	1.796 (0.491–4.792)	0.949
Post-swallowing jitter (local)	0.242 (0.169–0.545)	0.333 (0.116–0.922)	0.281
Post-swallowing shimmer (local)	**1.485 (1.113–3.02)**	**2.479 (0.93–8.65)**	**0.008**
Post-swallowing HNR	23.076 (17.652–28.39)	20.505 (14.23-26.791)	0.094

*Note*. IwMS = individual with Multiple sclerosis; Min = minimum; Max = maximum; GUSS = Gugging Swallow Screening Test; F0 = fundamental frequency; SD F0 = standard deviation F0; HNR = harmonic-to-noise ratio

ROC analysis results regarding the discrimination of dysphagia/penetration risk by acoustic parameters in IwMS are given (Table 8). For pre-swallowing shimmer (local), the area under the curve was 73.1% and the cutoff value was 1.69. For post-swallowing shimmer (local), it was found to be 78.6% and 1.57. The area under the curve shows the statistical significance of the discrimination ability of the diagnostic test. Since the diagnostic test evaluated in our study was the risk of dysphagia/penetration in individuals, it was determined that the value found for pre- and post-swallowing had a moderate discrimination ability (70-80%).

**Table 8:** ROC Analysis Results for the Discrimination of Dysphagia/Penetration Risk by Shimmer

Risk Factor	AUC (95% CI)	Cut-off	*p*-value	Sensitivity (%)	Specificity (%)
Pre-swallowing shimmer (local)	0.731 (0.561-0.902)	> 1.685	0.031	65.5	80.0
Post-swallowing shimmer (local)	0.786 (0.618-0.954)	> 1.572	0.008	89.7	60.0

*Note*. AUC = area under curve; CI = confidence interval

**Fig. 1:**
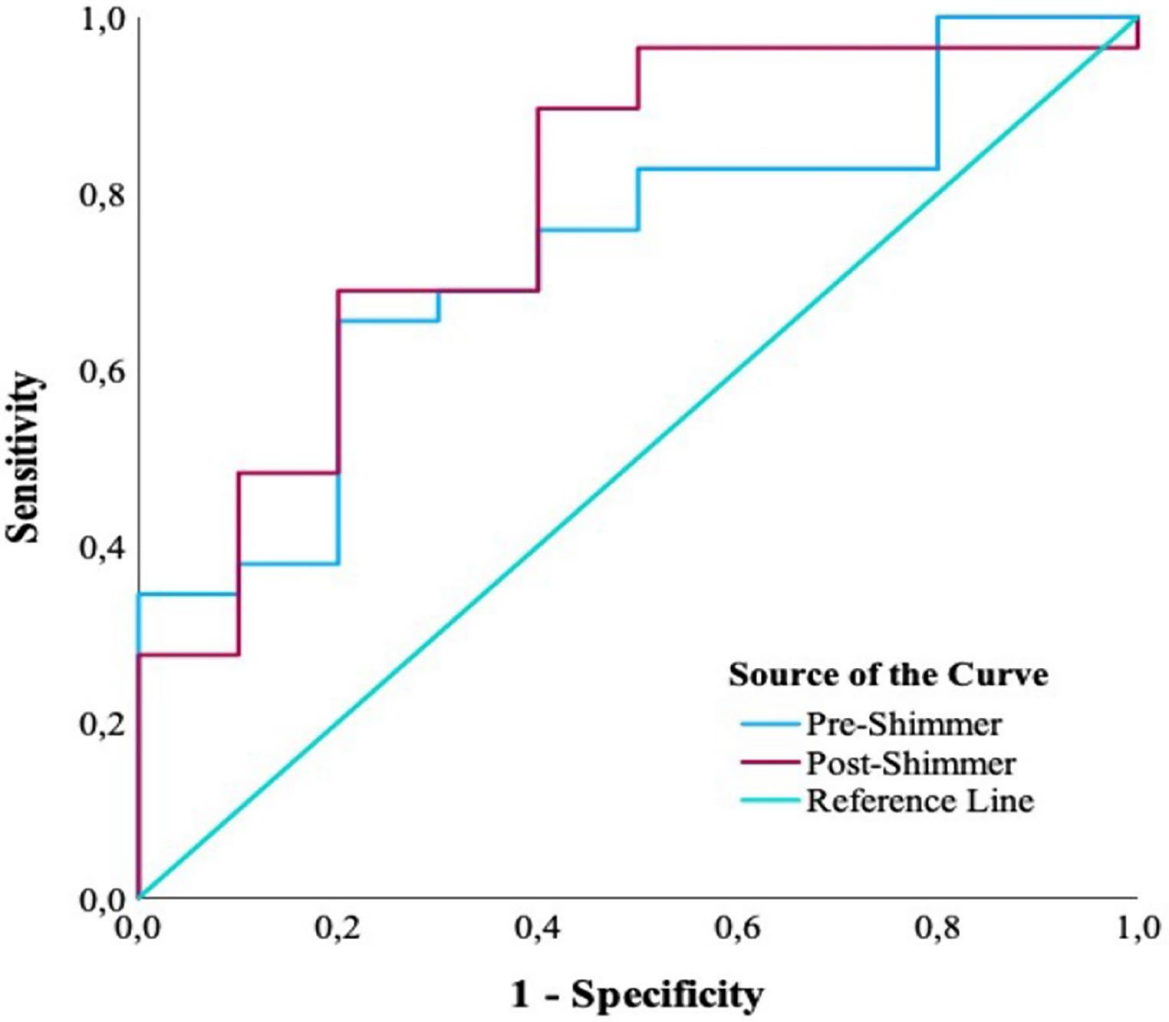
ROC curve for the discrimination of dysphagia/penetration risk by the shimmer (local) parameter. *Note*. ROC = receiver operating characteristic

The ROC curve made to examine the differential effect of the shimmer (local) parameter pre- and post-swallowing according to the risk of dysphagia/penetration in IwMS is shown (Fig. 1).

## Discussion

Perceptual assessment of changes in voice has been reported to be less sensitive and specific in determining the risk of penetration/aspiration [21, 48]. Technological innovations have made it common for clinicians to use acoustic analysis assessments in addition to perceptual assessment methods. Based on this information, acoustic voice analysis was used in this study. In order to identify voice parameters that could potentially predict the risk of aspiration/penetration, voice parameters were compared with DYMUS and GUSS scores and the correlations between the measurements were examined.

According to our findings regarding the first research question, the VHI-10 scores related to voice and the DYMUS scores related to swallowing were significantly higher in IwMS than in HI. Similarly, the GUSS scores of the MS group were significantly lower than those of the healthy group. At this stage, a significant difference was found between the two groups in all parameters except F0 and standard deviation F0 (SD F0). The lack of difference in the groups’ F0 and SD F0 parameters may be explained by the similar distribution of the groups in terms of age and gender. Considering the average age of both groups, the fact that the participants were young adults may explain this situation. The F0 value is often used to check whether a voice analysis is accurate, because the parameters obtained from acoustic analysis are based on a calculation of the periodicity of the signal wave [21]. Based on the correct measurement of this voice parameter, other parameters can be analyzed. Differences in other parameters can be attributed to the impaired coordination in the voice and swallowing muscles as a result of damage to the corticobulbar pathway in MS. In addition, motor and sensory deficits occur in MS due to widespread effects extending from the spinal cord to cortical structures. Therefore, IwMS may be expected to exhibit a different profile than HI in voice and swallowing functions [9]. However, this cannot be generalized to every IwMS and every type of MS. In this study, pre-test data regarding voice and swallowing evaluations in IwMS and HI were described. Additionally, a procedure regarding swallowing was performed, and these data were compared with pre- and post-swallowing measurements. For post-swallowing, no difference was found between the IwMS and HI groups in terms of F0, SD F0, or jitter (local), while a significant difference was found in terms of shimmer (local) and HNR. The significant difference in these two parameters may be explained by the decline in motor performance after a series of events for these functions in IwMS.

The findings regarding the second question of the research involves the relationships between the scores obtained from the three scales related to voice and swallowing, in other words, the possible connections between the variables. A positive correlation was found between the VHI-10 and the DYMUS in IwMS and HI groups, a finding that supports the literature indicating that voice and swallowing are highly intersecting functions. No significant correlation was found between the GUSS and the VHI-10 in either group. This may be because the VHI-10 is a self-reported scale, whereas the GUSS is a bedside swallowing screening performed by clinicians. Finally, there was a low negative correlation between the GUSS and DYMUS in the group with IwMS. This can be interpreted as reflecting the fact that the expressions of the items in the two scales differ in terms of positivity/negativity and the fact that the scores obtained from the two swallowing scales in MS have reciprocal consistency. The absence of this significant relationship in the healthy group can be explained by the fact that this group consists of HI who did not exhibit any profile related to swallowing. In this set of findings, the relationships between voice and swallowing scales and pre- and post-swallowing voice parameters were also analyzed. A relationship was identified between DYMUS and pre-swallowing HNR parameters in IwMS. There was a relationship between GUSS and post-swallowing shimmer (local) and pre-swallowing jitter (local) and HNR parameters in IwMS. This may be because the risk of dysphagia and changes in the voice coincide in IwMS. Since the structures of the voice and swallowing are anatomically related and close, a problem that may arise in relation to one may affect the other. The correlation in the shimmer (local) parameter only post-swallowing may be attributable to the fact that the material in the vocal fold causes instantaneous changes in amplitude. A correlation between GUSS and the post-swallowing HNR parameter was found in IH. However, no significant differences were found for this parameter pre- and post-swallowing. The correlation between shimmer (local) after swallowing and GUSS and the difference in the shimmer (local) before and after swallowing suggest that the combination of GUSS and shimmer (local) parameter may be useful in clinical evaluation.

In the findings regarding the third and fourth questions of the study that is consistent with the observation of a significant difference between the two groups in terms of shimmer (local) and HNR in the post swallowing is a significant difference within the IwMS in the same parameters between the pre- and post-swallowing. A change in in voice that reflect high-frequency noise may indicate a risk of aspiration [22]. This is in line with the findings of this study, which showed that the mean F0 did not change significantly but the HNR decreased significantly. However, no significant difference was found in any parameter in the healthy group. Therefore, it is important to compare pre-test and post-test acoustic voice parameters according to GUSS and DYMUS scores in IwMS. These findings reveal that the shimmer (local) parameter is significantly different pre- and post-swallowing in the IwMS. This sudden change in pre- and post-swallowing voice parameters in IwMS may have occurred due to aspiration/penetration. Changes in shimmer are indicative of irregularities in amplitude [22]. Abnormalities in amplitude and periodicity can occur as the bolus changes the pattern of vocal fold movements. Further investigation of whether this change in acoustic parameters is due to the presence of an ingested bolus in the vocal folds or elsewhere in the vocal tract may be instructive for clinical applications.

The results in the literature related to our findings are diverse, but there are remarkable intersections in some parameters. In a study of individuals with a cerebrovascular accident, general medical illness, or laryngeal disease with and without aspiration risk, Ryu et al. (2004) no significant difference was found in the F0 parameter between the high and low aspiration risk group as a result of the videofluoroscopic swallowing study (VFSS). In the present study, differences were found between the two groups before and after VFSS in the parameters of shimmer percentage, VTI, NHR, and RAP. It was reported that there was a significant increase in these parameters in the high risk group for aspiration after VFSS [22]. Chang et al. (2012) used similar methods and case groups to those of Ryu et al. but reported observing no significant change in voice parameters [49]. They state that the evaluated voice parameters could not identify the risk of aspiration/penetration. Jin Song et al. (2022) in their study using VFSS with 55 participants with suspected dysphagia, reported a significant relationship between shimmer values and silent aspiration. Additionally, in this study, the authors reported that it is possible to predict the distinction between penetration and aspiration by using voice parameters [50]. However, since no instrumental measurement was made in swallowing evaluation in our study, such an interpretation cannot be made. In the literature, this issue has been studied in various diseases. Kang et al. (2018), studying a stroke, medical disease, head and neck cancer, degenerative disease, and geriatric population, found that the RAP parameter decreased significantly in the aspiration risk group after videofluoroscopic swallowing observation compared to the low-risk group [21]. They also reported that the jitter parameter increased in the aspiration risk group post-swallowing, but the difference was not significant. De Bruijn et al. investigated the relationship between acoustic voice parameters and swallowing function in patients with oral or oropharyngeal cancer and found that the acoustic parameters were not associated with swallowing function [23]. It is thought that the reasons for the changes seen in various parameters in our study and other studies in the literature may be the heterogeneity of the case groups, different acoustic analysis methods used and diverse age groups of the participants. In addition, variation in bolus consistencies and volumes used in the swallowing evaluation may explain differences in previous findings.

The results of the fifth and sixth study questions demonstrate that, following the acquisition of all these correlations and comparisons, we used the advanced statistic of ROC analysis to examine whether the shimmer (local) parameter is of diagnostic value for dysphagia/penetration. In the result, the area under the curve (AUC) for pre-swallowing shimmer (local) is 73.1%, and the cutoff value is 1.69. For post-swallowing shimmer (local), the respective values are 78.6% and 1.57. The AUC shows the statistical importance of the discriminative ability of the diagnostic test. In our study, the value shown in the diagnostic test used to identify individuals with dysphagia/penetration risk had a moderate discriminative ability (70—80%) for pre- and post-swallowing. In the clinical setting for IwMS, pre-swallowing shimmer (local) values of 0.0–1.69 and post-swallowing shimmer (local) values of 0.0–1.57 may be considered normal for dysphagia/penetration risk, but higher values may indicate a risk for dysphagia/penetration. Residue in the vocal folds may cause irregularities in the vocal fold amplitude and therefore may cause changes in the shimmer (local) value.

This study is a theoretical descriptive study. However, although its applicability is debatable, it also has clinical implications. It has been reported in the literature that the presence/absence of wet voice can be examined in clinical evaluation to determine the risk of penetration/aspiration. However, it has also been reported that wet voice may not be a good clinical predictor of the risk of aspiration during swallowing [24, 48, 51].

Our study first shows that the inclusion of acoustic voice analysis measurements pre- and post-swallowing provides a complementary tool to bedside swallowing assessment in IwMS that may be helpful in predicting the risk of dysphagia/penetration. Among the acoustic voice parameters, especially the change in shimmer (local) value should be taken into consideration. Second, it suggests that acoustic analysis may be useful for clinicians who cannot access instrumental swallowing assessment tools in the clinic, as it is easy to apply and costs little. Finally, the study used both subjective and objective methods to evaluate voice and swallowing and discusses in detail the relationship between these functions in individuals. Thus, our findings may guide the evaluation and therapy steps. The first limitation of our study is that instrumental imaging methods were not used in the evaluation of either voice or swallowing. Instrumental evaluation is needed to provide detailed interpretations on whether the shimmer and HNR value are indicative of residue or aspiration/penetration in clinical practice. However, in our study, the GUSS with high sensitivity and specificity was employed in our investigation to assess the dysphagia risk [33]. The second is that loudness and/or other acoustic parameters were not included in the study. VTI and RAP parameters that may be related to aspiration and penetration were not analysed. These parameters can also be included in future research.

## Conclusions and Implications

This study’s first conclusion is that IwMS as a group can be associated by shimmer (local), and HNR voice parameters, voice handicap, and dysphagia and penetration risk. All the measurements largely confirm one another. The second conclusion is that shimmer (local) has a diagnostic value for dysphagia-penetration risk at above > 1.69 pre-swallowing and above > 1.57 post-swallowing. Of course, this study has no direct clinical implications; the basic research aspect is more prominent, as the clinical standard procedures for aspiration/penetration are clear. However, taking into account acoustic measurements as well as standard procedures in swallowing evaluation may further strengthen diagnostic decisions. As findings in this area emerge in future research, they will be reflected in clinical practice and diagnostic processes will be supported with low-cost, portable, and easy-to-use acoustic software. It is therefore hoped that, in the future, practical procedures, such as examining voice parameters, will be reflected in innovations (e.g., shimmer-specific apps for smartphones) to predict dysphagia-penetration risk. In addition, for a more accurate and reliable swallowing assessment, the use of objective instrumental imaging methods is recommended as well as acoustic analysis is recommended again after each viscosity of bolus. The agreement between shimmer and instrumental measurements could be examined with statistics such as the Bland-Altman plot.
